# Dietary intake of fruits and vegetables, polyunsaturated fats, and fish and positive psychological well‐being in older adults: A cross‐sectional analysis of the English Longitudinal Study of Ageing (ELSA)

**DOI:** 10.1111/bjhp.70022

**Published:** 2025-09-15

**Authors:** Pepper Thanaporn Theeraoat, Ruth A. Hackett, Joseph Chilcot, Andrew Steptoe

**Affiliations:** ^1^ Department of Psychology, Institute of Psychiatry, Psychology and Neuroscience King's College London London UK; ^2^ Department of Behavioural Science and Health University College London London UK

**Keywords:** ageing, diet, nutrition, well‐being

## Abstract

**Objectives:**

Research has linked diet to negative psychological states, but its influence on positive psychological well‐being remains understudied. This study assessed the association between dietary intake of fruits and vegetables (F&V), polyunsaturated fats (PUFAs), and fish on three domains of positive well‐being: eudemonic, happiness, and life satisfaction in middle‐aged and older adults.

**Design:**

A cross‐sectional analytical sample of 3013 participants from Wave 9 (2018/19) of the English Longitudinal Study of Ageing (ELSA).

**Methods:**

Multivariate linear regression assessed the association between diet and positive psychological well‐being, adjusted for covariates including total energy intake, age, gender, ethnicity, wealth, education, living alone, social isolation, limiting long‐standing illness, and depressive symptoms.

**Results:**

In minimally adjusted models, F&V and fish intake were positively associated with all three domains of well‐being, while PUFAs intake was positively associated with eudemonic well‐being and happiness, but not life satisfaction. The positive associations between F&V intake and eudemonic well‐being, and between fish intake and happiness, remained significant in all models (*β* = .043, 95% CI [.037, .212], *p* = .005; *β* = .033, 95% CI [.011, .243], *p* = .032, respectively), whereas others became non‐significant after adjusting for certain covariates.

**Conclusions:**

Diet may be associated with positive psychological well‐being in middle‐aged and older adults. Increasing dietary intake of F&V, PUFAs, and fish could support well‐being and may be encouraged through public or private initiatives aimed at making healthy diets accessible and affordable. Longitudinal studies are needed to clarify the influence of diet on well‐being over time.

**Statement of Contribution**

**What is already known on this subject?**

- Existing evidence shows a healthy diet can improve negative psychological states, especially depression.
- There is limited research on whether dietary intake can promote positive psychological well‐being.

**What does this study add?**

- Fruits and vegetables, polyunsaturated fats, and fish consumption may promote positive well‐being.
- The link between diet and well‐being was attenuated after adjustment for covariates.
- Several associations between diet and positive well‐being are not independent of depressive symptoms.

## INTRODUCTION

The global demographic is rapidly shifting towards an ageing population, with the proportion of people aged over 60 expected to double from 12% in 2015 to 22% in 2050 (WHO, 2022). As ageing is often accompanied by health decline that can impact quality of life, it is important to identify factors that promote health and well‐being. Diet is one modifiable risk associated with physical and mental health outcomes (Dash et al., 2016; Ding et al., 2022; Marx et al., 2017; Meegan et al., 2017), with studies suggesting that dietary improvement is an accessible and efficacious treatment strategy for major depression (Jacka et al., 2017). The benefits of fruits and vegetables (F&V), polyunsaturated fats (PUFAs), and fish consumption on physical and mental health have been widely established. However, the role of diet in increasing positive psychological states remains understudied.

The UK dietary guidelines recommended consumption of at least five portions of F&V daily (a portion = 80 g or 3 oz) and at least two portions of fish weekly (a portion = 140 g or 4.9 oz; Public Health England, 2016). Total fat intake should not exceed 35% of daily energy, with 6.5% being PUFAs, a type of fat that includes omega‐3 and omega‐6 fatty acids, essential for cell, nerve, and brain functions. However, the majority of the population does not meet the dietary recommendations (GOV.UK, 2023).

### Diet and negative psychological states

Existing studies have linked diet to negative psychological states. A cross‐sectional UK Biobank Study (*N* = 502,494 middle‐aged adults) linked higher F&V and fish intake with lower psychological symptomatology (e.g., miserableness, irritability, etc.; Hepsomali & Groeger, 2021). Systematic reviews and meta‐analyses suggest the benefits of fish and PUFAs intake throughout the lifespan, including decreased risk of depression (Bozzatello et al., 2019; Pusceddu et al., 2016). Plausible underlying mechanisms for the association between F&V intake and well‐being include changes in the gut microbiome that modulate brain chemistry, reduction in oxidative stress and inflammation, and increase in neurotransmitter production (Fatahi et al., 2021; Sivaprakasam et al., 2016; Swann et al., 2020; Taylor et al., 2020). For PUFAs and fish, the modulation of neurotransmitter levels (e.g., dopamine, serotonin), promotion of neurogenesis and hippocampal brain‐derived neurotrophic factor (BDNF), mitigation of the hypothalamic pituitary adrenal (HPA) axis activation in response to stressors, and reduction of oxidative stress, neuroinflammation, and neurodegeneration were suggested (Brito et al., 2019; Healy‐Stoffel & Levant, 2018; Pusceddu et al., 2016; Su et al., 2015; Sublette et al., 2014; Zhou et al., 2022). However, whether a good diet rich in F&V, PUFAs and fish can improve positive psychological states remains under‐researched.

### Diet and positive psychological well‐being

The conceptualization of positive well‐being (synonymous with subjective/mental well‐being) surpasses the absence of mental ill‐health and includes feeling and functioning well (Huppert, 2009; Ruggeri et al., 2020). It is a multidimensional construct with three domains: (1) Eudemonic well‐being: referring to a sense of purpose, control, and meaning of life, positive relationships, and personal growth; (2) Hedonic or affective well‐being: involving positive emotions including happiness, contentment, and pleasure; (3) Evaluative well‐being: reflecting overall life satisfaction.

### Fruit and vegetable intake and positive well‐being

Most diet and well‐being research has examined F&V intake. Positive associations were found in middle‐aged and older adults. Cross‐sectional data from the Welsh Health Survey (2007–2010), the Scottish Health Survey (2008), and the Health Survey of England (2008) indicated that individuals who consumed more F&V had greater happiness, life satisfaction, and overall well‐being, with the best results evident at 7–8 portions daily (Blanchflower et al., 2013).

In addition to cross‐sectional findings, longitudinal studies provide further insights into the relationship between diet and well‐being. Analyses of three waves of the UK Household Longitudinal Survey (2010–2017), controlling for diet and health behaviours, showed that increases in both quantity and frequency of F&V consumption enhanced mental well‐being and life satisfaction (Ocean et al., 2019). Using large Australian household data (HILDA 2007–2009), positive longitudinal associations over 2 years of follow‐up were found between F&V intake and happiness and life satisfaction among individuals aged 15–93, suggesting that raising F&V intake would enhance well‐being (Mujcic & Oswald, 2016). Prospective analyses of well‐being on lagged F&V consumption demonstrated that F&V intake in the current year was predictive of happiness and life satisfaction in the future, even after controlling for current well‐being and covariates. No evidence for reverse causality was observed as happiness and life satisfaction did not predict future F&V consumption. Higher F&V consumption was found to improve mental health in an analysis of middle‐aged and older adults from 11 European countries, using the Survey of Health, Ageing and Retirement in Europe data (2011, 2013; Gehlich et al., 2020). Similar results were also found in non‐Western countries (Gehlich et al., 2019).

### PUFAs, fish intake and positive well‐being

While PUFAs and fish intake have been linked to poor mental health (Bozzatello et al., 2019; Brito et al., 2019; Rao et al., 2008), research on positive well‐being is limited. In a small randomized crossover‐design study, positive affect was significantly higher when participants consumed a higher PUFAs diet compared with when they had lower PUFAs or a control diet (Lindseth & Petros, 2016). Positive evidence in older adults exists, but supplements were examined instead of dietary intake. In a randomized control trial study, an increase in positive mood was found among elderly Japanese men in the PUFAs supplementation group, compared to the placebo group (Tokuda et al., 2017).

### Direction of the diet and well‐being relationship

The direction of the relationship between diet and well‐being is uncertain, although it has been proposed to be bidirectional (Trudel‐Fitzgerald et al., 2019). In the aforementioned Australian sample, higher F&V intake predicted higher future life satisfaction and happiness, even after accounting for initial well‐being and covariates, but well‐being did not predict future F&V intake (Mujcic & Oswald, 2016). In contrast, previous work in the English Longitudinal Study of Ageing (ELSA) cohort suggested well‐being (measured with 17 items from the Control, Autonomy, Satisfaction, Pleasure Scale) might be a precursor to higher F&V consumption. Longitudinal associations across 7 years showed a positive association between baseline well‐being and baseline F&V consumption, and despite a general decline in F&V intake across time, individuals with higher baseline well‐being had slower declines (Boehm et al., 2018). Fish and PUFAs were not examined in this study.

### Factors influencing diet and well‐being

Several factors are known to influence diet and well‐being including demographic, socio‐economic, social, and physical and mental health factors. Firstly, ageing has been found to influence dietary intake. Older adults tend to consume more F&V (Drewnowski & Shultz, 2001). However, low nutrient density diets were also common among this age group. Consistent evidence links higher socio‐economic status (wealth, income, education) with better diet including higher F&V consumption (Alkerwi et al., 2015; Foroozanfar et al., 2022), and suggests the link between social factors (e.g., isolation, living alone) and diet among middle‐aged and older adults (Bloom et al., 2017; Boulos et al., 2017; Ramic et al., 2011; Walker & Beauchene, 1991). Moreover, physical and mental health problems can undermine diet quantity and quality. For instance, individuals with illnesses may have dietary restrictions that determine how much and what foods they can consume, and disabilities can impact eating and food preparation (van den Heuvel et al., 2019). As mentioned above, low F&V, PUFAs, and fish intake have been associated with depression. Finally, positive well‐being is suggested to be higher at older age (Stone et al., 2010) and is associated with better economic circumstances (Kahneman & Deaton, 2010), social connections, and mental and physical health, including survival and longevity (Martín‐María et al., 2017; Steptoe et al., 2005, 2015; Steptoe & Lassale, 2018).

In the ELSA cohort, lower F&V consumption was more commonly observed in socially isolated adults compared to their less isolated counterparts (Ishikawa et al., 2017; Kobayashi & Steptoe, 2018; Whitelock & Ensaff, 2018). In Wave 9, demographic differences in nutritional intake were observed. Women were found to consume more F&V than men, and wealthier participants had higher fibre and lower fat intake. Higher wealth groups also reported greater consumption of F&V, nuts and seeds, and fish compared with lower wealth groups (Di Gessa et al., 2020).

### Conclusion and study aim

In summary, previous research on diet and mental health has focused on negative psychological states. Emerging evidence on positive well‐being and F&V, PUFAs and fish intake suggest that these dietary factors may support not just the alleviation of negative states but also in promotion of positive mental health and well‐being. While previous studies have included demographic, socio‐economic, and physical health factors as covariates, none has adjusted for negative psychological states such as depressive symptoms. Different studies in the field have focused on different aspects of well‐being. However, associations with all three aspects of well‐being have not been assessed simultaneously in relation to F&V, PUFAs, and fish intake.

The aim of this study was to examine the association between diet and positive well‐being among middle‐aged and older adults in England, using data from the nationally representative ELSA study. Specifically, nutrition data introduced in Wave 9 was used. We hypothesized that higher dietary intake of F&V, PUFAs, and fish will be associated with higher eudemonic well‐being, happiness, and life satisfaction, and these associations will be attenuated after adjustment for covariates, including demographic, social, and health factors.

## METHOD

### Study sample

ELSA is a representative sample of adults aged 50 and over living in England (see Steptoe, Breeze, et al., 2013 for details). The current study uses data from ELSA Wave 9 (2018/19) which was the most recent wave of data collection at the time of analysis. A total of 5068 men and women aged over 50 participated in this wave. Ethical approval for the study was obtained from London Multicentre Research and Ethics Committee (MREC/01/02/91) and all participants provided fully informed consent.

### Measures

#### Dietary intake

F&V, PUFAs, and fish intake were measured through The Oxford WebQ (Liu et al., 2011), a 24‐h recall questionnaire assigned to complete on two randomly allocated days (see Steptoe et al., 2024 for details). The scale is suitable for large‐scale prospective studies and has been validated in middle‐aged and older adults (Galante et al., 2016; Greenwood et al., 2019; Perez‐Cornago et al., 2021).

#### Positive well‐being

Eudemonic well‐being was assessed using 15 items from the CASP‐19 scale (Hyde et al., 2003), covering subscales including control (6 items), autonomy (5 items), and self‐realization (4 items). Items were rated from 0 (never) to 3 (often); total scores were then summed (0–45); higher scores indicate better well‐being. The scale has been widely used in the study of ageing and has been validated in over 20 countries with adequate psychometric properties (Kim et al., 2015).

For hedonic well‐being, the question “*Overall, how happy did you feel yesterday?”* from the Office for National Statistics well‐being survey was answered on a scale of 0 (not at all) to 10 (completely). This method increases the possibility of inducing a hedonic response instead of an evaluative response (Steptoe et al., 2015).

Lastly, life satisfaction was measured using the 5‐item Satisfaction with Life Scale (Diener et al., 1985). Items were rated from 0 (strongly disagree) to 6 (strongly agree). Total scores are summed (0–30); higher scores indicate greater satisfaction. The scale has been widely used and has been validated in diverse population groups (López‐Ortega et al., 2016; Magyar‐Moe, 2009; Weber et al., 2015).

#### Covariates

Ten potential confounding variables were adjusted for in the analyses, all were assessed at Wave 9. As in other ELSA dietary analyses (Steptoe et al., 2024), total energy intake was taken into account and measured as kilocalories consumed per day. Age was measured in years. Gender was measured as male or female. Ethnicity was measured as White or non‐White. Wealth was indexed as total household wealth across the entire Wave 9 sample, resulting in five equal‐sized quintiles, ranging from 1 (lowest) to 5 (wealthiest). For education, participants were categorized into three groups according to their highest qualification: Less than O‐level or equivalent (coded as 0), O‐level or equivalent (1), and higher than A‐level (2).

Whether respondents were living alone was coded in binary (alone/not alone). Social isolation was assessed using the Social Isolation Index (Steptoe, Shankar, et al., 2013), developed for ELSA and previously linked to health outcomes in this sample (Hackett et al., 2020; Rafnsson et al., 2020). Total score ranges from 0 to 4; higher scores indicate greater isolation.

Limiting long‐standing illness was assessed by asking whether respondents have any illness, disability or infirmity that affected them over at least 6 months and whether that limits their activities. Responses were binary (yes/no).

Finally, depressive symptoms were measured using the eight‐item Centre for Epidemiologic Studies Depression Scale (CES‐D; Radloff, 1977), a widely used instrument with good validity among older adults (APA, 2011; Lau et al., 2022; Steffick, 2000). Responses were binary (yes/no). Total scores are summed (0–8); higher scores indicate greater depression. A score of 4 and above denoted significant depression in previous studies (Demakakos et al., 2010; White et al., 2016).

### Statistical analysis

The exposures of F&V (daily portion), PUFAs (% from total energy intake), and fish (daily portion) intake were skewed and were therefore statistically adjusted to a normal distribution. The SPSS square root function was used for F&V and PUFAs, whereas fish was adjusted into binary categories: no (0) or some (≥1) fish consumed.

Multivariable linear regression, adjusting for covariates, was conducted to assess the associations between each of the dietary variables and the three positive well‐being outcomes.

Associations are presented as standardized regression coefficients with *p* values and 95% confidence interval as well as coefficient of determination for the dietary variable. For each association, five models were tested. Model 1 adjusted for total energy intake, age, and gender. Demographic indicators (wealth, education, ethnicity) were added in Model 2. Social factors (living alone, isolation) were added in Model 3. Physical health (limiting long‐standing illness) was added in Model 4, and mental health (depressive symptoms) was added in Model 5 to explore whether associations between diet and positive well‐being are independent of negative psychological states. Data were analysed using IBM SPSS 27.0.

## RESULTS

### Sample characteristics

**FIGURE 1:**
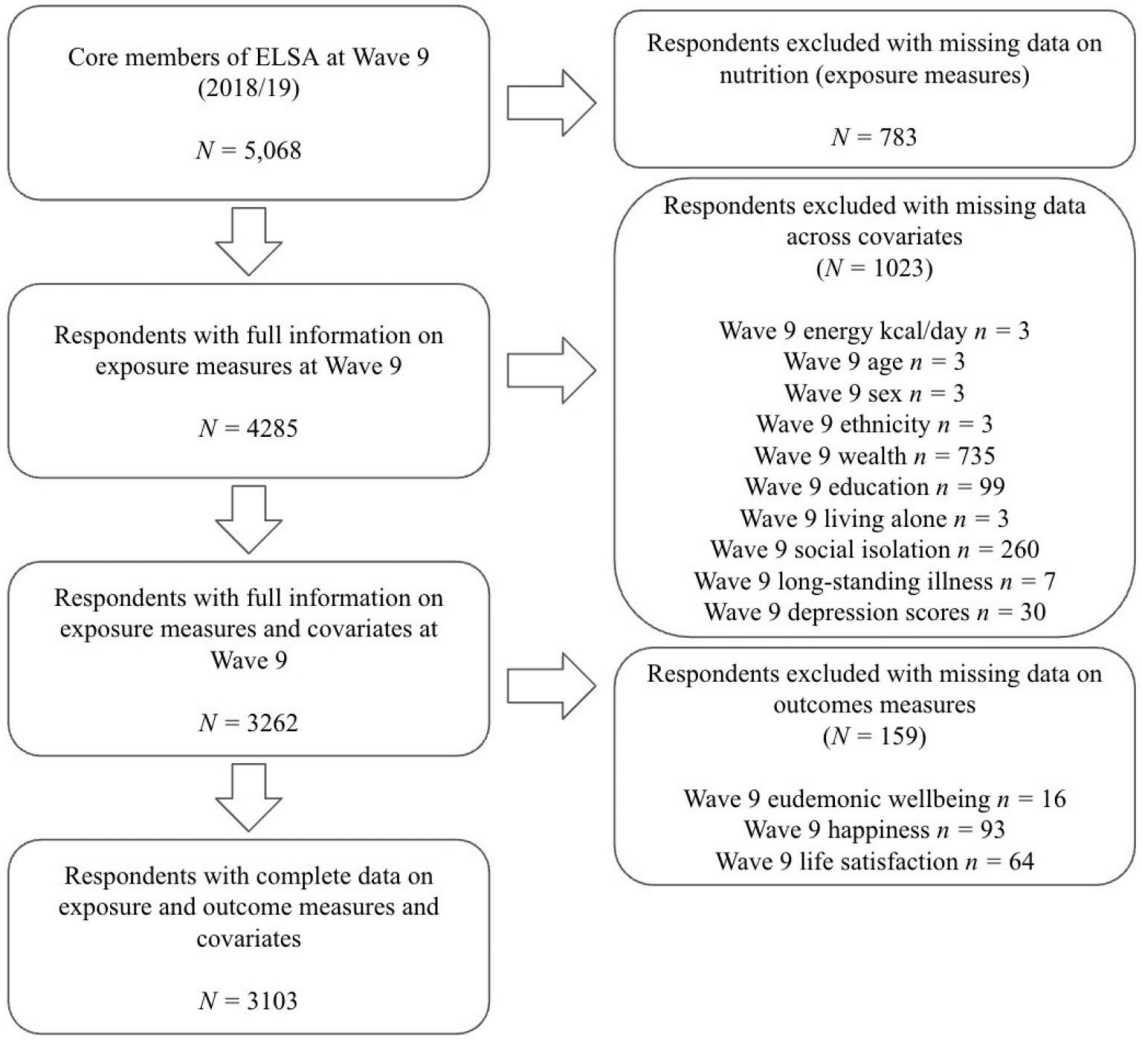
Flowchart of participants excluded and included in the analysis. ELSA, English Longitudinal Study of Ageing.

The characteristics of the sample can be found in Table 1. After excluding missing data (*see* Figure 1), the final sample consisted of 3103 individuals (53.8% women). The age of participants in the study ranged from 54 to 99 years (*M* = 69.33 years, SD = 7.23). Most respondents were of White ethnicity (97.8%), in the highest wealth quintile (30.1%), and were educated up to high school (49.7%). The majority of respondents did not live alone (79.0%) and were not socially isolated (*M* = 0.78, SD = 0.87). Most reported having no limiting long‐standing illness (70.0%) and had low depressive symptoms scores (*M* = 1.07, SD = 1.56).

**TABLE 1:** Sample characteristics and associations with key variables (*N* = 3103).

	*n* (%) or *M* (SD)	F&V	PUFAs	Fish	Eudemonic wWell‐being	Happiness	Life satisfaction
Energy kcal/day	2042.86 (624.81)	.252**	.184**	.021	.006	−.007	.040*
Age (years)	69.33 (7.23)	.063**	−.043*	.060**	−.071**	.060**	.033
Gender (% women)	1668 (53.8%)	.133*	.030	.035*	.000	−.021	−.054**
Ethnicity (% White)	3036 (97.8%)	−.025	.019	−.002	−.020	−.024	−.023
Wealth							
Quintile 1	302 (9.7%)	.142**	.000	.106**	.266**	.139**	.231**
Quintile 2	399 (12.9%)						
Quintile 3	636 (20.5%)						
Quintile 4	831 (26.8%)						
Quintile 5	935 (30.1%)						
Education							
<O‐level or equivalent	566 (18.2%)	.110**	−.022	.044*	.119**	.022	.076**
=O‐level	995 (32.1%)						
>A‐level	1542 (49.7%)						
Living alone (% not alone)	2451 (79.0%)	.035*	−.026	.023	−.087**	−.096**	−.226**
Social isolation (mean)	.78 (87)	−.113**	−.017	−.073**	−.148**	−.097**	−.125**
Long‐standing illness (% no)	2172 (70.0%)	−.057**	−.023	−.056**	−.400**	−.212**	−.231**
Depressive scores (mean)	1.07 (1.56)	−.075**	−.044*	−.043*	−.498**	−.512**	−.454**
F&V (mean)	2.03 (.80)		.064**	.083**	.111**	.069**	.062**
PUFAs (mean)	2.49 (.46)			.120**	.046*	.038*	.023
Fish							
None	1782 (57.4%)				.061**	.066**	.046*
Some	1321 (42.6%)						
Eudemonic well‐being (mean)	11.06 (2.28)					.603**	.663**
Happiness (mean)	7.88 (1.90)						.582**
Life satisfaction (mean)	21.61 (5.76)						

*Note*: *p < .05, **p < .01. Scale Ranges: Eudemonic Wellbeing: 0–45; Happiness: 0–10; Life Satisfaction: 0–30; Depressive Symptoms: 0–8. Higher scores indicate greater well‐being (for well‐being measures) and more severe symptoms (for depressive symptoms).

Abbreviations: F&V, fruit and vegetables; PUFAs, polyunsaturated fats.

On average, respondents consumed 2042.86 kilocalories (SD = 624.81) and two portions of F&V daily (SD = 0.80). PUFAs represented 2.49% of participants' overall daily energy intake (SD = 0.46). More than half of the respondents (57.4%) reported no fish consumption on the days of data collection, with the rest (42.6%) reporting having eaten some fish (≥1). Well‐being was relatively high in the sample. Average eudemonic well‐being was 11.06 (SD = 2.28; Min. = 1.33, Max. = 15). Happiness ranged from 0 to 10, with an average score of 7.88 (SD = 1.90). Life satisfaction ranged from 0 to 30, with an average score of 21.61 (SD = 5.76).

### Intercorrelations

Overall, there was no collinearity problem as the correlations did not exceed *r* = .70, with the highest intercorrelation of *r* = .66 found between eudemonic well‐being and life satisfaction variables (See Table 1). There were small, positive correlations among the three dietary intakes and strong positive correlations among the three well‐being domains. All three diet variables were weakly positively correlated with all three aspects of well‐being (i.e., higher intake, higher well‐being). There were several significant correlations between covariates and key variables. For instance, wealth and education were positively correlated with most key variables except PUFAs intake (and happiness in the case of education). Negative correlations were generally found for social isolation, long‐standing illness, and depressive symptoms. Ethnicity showed no significant associations.

### Multivariable linear regression results

#### Eudemonic well‐being

F&V intake remained significantly, positively associated with eudemonic well‐being in all models (*β* = .043, 95% CI [.037, .212], *p* = .005; see Table 2). However, PUFAs intake was positively associated with eudemonic well‐being until depressive symptoms scores were adjusted for in the final model (*β* = .023, 95% CI [−.030, .259], *p* = .120). Fish was positively associated with eudemonic well‐being up to Model 2 (*β* = .037, 95% CI [.012, .326], *p* = .035).

**TABLE 2:** Dietary intake and eudemonic well‐being regression results.

	Model 1 (Total energy intake, age, sex)	Model 2 (Ethnicity, wealth, education)	Model 3 (Living alone, social isolation)	Model 4 (Long‐standing illness)	Model 5 (Depressive symptoms)
F&V intake					
*β*	.128**	.086**	.082**	.067**	.043**
*R* ^2^	.018	.086	.098	.214	.353
CI 95%	[.261, .473]	[.142, .350]	[.131, .338]	[.095, .289]	[.037, .212]
PUFAs intake					
*β*	.043*	.045*	.045*	.039*	.023
*R* ^2^	.006	.082	.094	.211	.352
CI 95%	[.035, .392]	[.053, .397]	[.051, .392]	[.037, .355]	[−.030, .259]
Fish intake					
*β*	.065**	.037*	.032	.017	.010
*R* ^2^	.008	.081	.093	.210	.352
CI 95%	[.139, .464]	[.012, .326]	[.‐.007, .305]	[−.068, .224]	[−.086, .178]

* *p* < .05.

** *p* < .01.

#### Happiness

Fish intake remained independently associated with happiness in the fully adjusted model (*β* = .033, 95% CI [.011, .243], *p* = .032; see Table 3). F&V and PUFAs were positively associated with happiness until depressive symptoms scores were added to the model.

**TABLE 3:** Dietary intake and happiness regression results.

	Model 1 (Total energy intake, age, sex)	Model 2 (Ethnicity, wealth, education)	Model 3 (Living alone, social isolation)	Model 4 (Long‐standing illness)	Model 5 (Depressive symptoms)
F&V intake					
*β*	.081**	.065**	.064**	.055*	.027
*R* ^2^	.009	.024	.035	.072	.278
CI 95%	[.104, .281]	[.065, .243]	[.063, .241]	[.045, .219]	[−.013, .141]
PUFAs intake					
*β*	.046*	.047*	.045*	.042*	.022
*R* ^2^	.005	.023	.033	.071	.278
CI 95%	[.043, .340]	[.046, .341]	[.038, .331]	[.029, .316]	[−.036, .217]
Fish intake					
*β*	.064**	.051**	.050**	.041*	.033*
*R* ^2^	.007	.023	.034	.071	.279
CI 95%	[.111, .381]	[.062, .331]	[.058, .326]	[.027, .290]	[.011, .243]

* *p* < .05.

** *p* < .01.

#### Life satisfaction

F&V intake was positively associated with life satisfaction until long‐standing illness was adjusted for in Model 4 (*β* = .028, 95% CI [−.053, .459], *p* = .121; See Table 4). No significant association was found between PUFAs and life satisfaction. The positive association between fish intake and life satisfaction was significant only in Model 1 (*β* = .045, 95% CI [.117, .937], *p* = .0112).

**TABLE 4:** Dietary intake and life satisfaction regression results.

	Model 1 (Total energy intake, age, sex)	Model 2 (Ethnicity, wealth, education)	Model 3 (Living alone, social isolation)	Model 4 (Long‐standing illness)	Model 5 (Depressive symptoms)
F&V intake					
*β*	.067**	.034*	.036*	.028	.005
*R* ^2^	.007	.055	.097	.132	.262
CI 95%	[.218, .757]	[−.020, .514]	[.001, .523]	[−.053, .459]	[−.198, .276]
PUFAs intake					
*β*	.022	.023	.017	.015	−.001
*R* ^2^	.004	.055	.096	.132	.262
CI 95%	[−.176, .725]	[−.151, .727]	[−.211, .649]	[−.238, .605]	[−.403, .375]
Fish intake					
*β*	.045*	.022	.024	.015	.016
*R* ^2^	.005	.055	.096	.132	.262
CI 95%	[.117, .937]	[−.146, .658]	[−.162, .671]	[−.209, .564]	[−.256, .457]

* *p* < .05.

** *p* < .01.

## DISCUSSION

The aim of this study was to examine the association between diet and positive psychological well‐being in middle‐aged and older adults in England, taking into account possible confounding factors. The results suggest that F&V, PUFAs, and fish consumption may promote positive well‐being, and these associations were attenuated after adjustment for covariates. F&V was the strongest predictor for eudemonic well‐being and life satisfaction, and fish was the strongest predictor for happiness. Nevertheless, several relationships between diet and positive well‐being were not independent of depressive symptoms, except for the positive relationships between (1) F&V and eudemonic well‐being, and (2) fish and happiness, which remained robust independent of all covariates.

Key findings from univariate analysis indicate that higher dietary intake of F&V, PUFAs, and fish were related to higher well‐being and lower depressive symptoms. From descriptive statistics of this sample, middle‐aged and older adults in England typically had a high level of well‐being, and consumed less F&V, PUFAs, and fish than recommended by the UK dietary guidelines. Correlations showed that those who consumed more total energy per day also had higher intake of F&V and PUFAs, but no significant relationship was found with fish intake, which might be due to more than half of the respondents reporting no consumption of fish on the randomly allocated 2 days that data were collected. Among older participants in the sample, higher intake of F&V and fish, but lower intake of PUFAs, was observed. While F&V and fish intake were higher among women, no robust relation was found between gender and PUFAs intake. Results partially supported the link between social factors and diet suggested by past research (Bloom et al., 2017; Boulos et al., 2017; Ishikawa et al., 2017; Kobayashi & Steptoe, 2018; Ramic et al., 2011). Socially isolated individuals had lower F&V and fish intake. However, those who reported living alone had slightly higher F&V intake. Arguably, living alone does not always translate to having poor social connections as one may live alone but still be in contact with others and have satisfactory social involvement. As expected, higher education and wealth were associated with better intake, except for PUFAs where no robust relations were found. This might be due to the amount of PUFAs intake that is too small for many covariates to detect an association with it.

For well‐being, life satisfaction was higher among those who consumed more total energy per day, but eudemonic well‐being and happiness did not vary systematically with how much total energy a person consumed. Eudemonic well‐being was found to be negatively associated with age, while happiness was positivity associated with age; age was not associated with life satisfaction. This may be due to the wide age range in the ELSA sample, as past research found large age differences in life satisfaction, whereby satisfaction increased from the age of 40 onwards, but sharply declined after 70 (Baird et al., 2010). In line with past findings that social productivity tends to decline with age, especially with retirement where occupations were a source of identity, self‐esteem, financial freedom, and social engagement opportunities, all of which contribute to eudemonic well‐being, and therefore can lead to reduction (Haslam et al., 2018; Wang, 2007). The result that happiness increased with age also supports the age‐related positivity effect in the literature, which is a well‐replicated empirical observation, referring to the cognitive processing of older adults that favours positive over negative information and therefore displays better well‐being (Kim & Barber, 2022; Reed & Carstensen, 2012). Gender was found to be unrelated to well‐being, except for life satisfaction which was lower among women. No robust relation was found between ethnicity and well‐being; however, this finding may be influenced by the small proportion of non‐White participants (2.2%) in this sample. Consistent with existing literature, higher wealth and education were linked to better well‐being, whereas living alone, social isolation, long‐standing illness and depressive symptoms seem to compromise well‐being. Higher education was related to higher eudemonic well‐being and life satisfaction, but not associated with happiness. This may be due to stress and responsibilities that come with education, potentially undermining daily or momentary affect.

The hypothesis that higher intake of F&V, PUFAs, and fish will be associated with higher positive psychological well‐being, and that the associations will be attenuated after adjustment for covariates was partially supported. Overall, positive relationships (i.e., higher intake, higher well‐being) were found except for between PUFAs and life satisfaction, and the strength of relationships was attenuated when adjusted for other factors, or became no longer robust when certain factors were considered, suggesting that the covariate contributed to a substantial part of the association between a particular diet and well‐being. Several associations between diet and positive well‐being were not independent of negative psychological states, as seen from the results that the relationships were no longer robust when depressive symptoms were taken into account. However, exceptions were evident for the positive relationships between (1) F&V and eudemonic well‐being, and (2) fish and happiness.

The first type of dietary intake investigated was F&V. Respondents with higher F&V intake reported higher eudemonic well‐being, happiness, and life satisfaction, with some variations. Higher F&V intake was associated with higher eudemonic well‐being even when all covariates were considered. However, it was no longer associated with happiness when depressive symptoms were taken into account, and neither with life satisfaction when long‐standing illness was considered. Consistent with the literature including large‐scale studies, F&V intake appears to promote positive well‐being to a certain extent, specifically happiness and life satisfaction (Blanchflower et al., 2013; Mujcic & Oswald, 2016; Ocean et al., 2019); none of the previous studies has examined eudemonic well‐being as the outcome nor have taken depressive symptoms into consideration. The current findings support the association between F&V intake and depression previously established in existing research. Despite some relationships not being independent of negative psychological states (e.g., F&V and happiness), the important finding here is that the link between F&V and eudemonic well‐being remained significant even after depressive symptoms were taken into account.

Next, PUFAs intake was positively associated with eudemonic well‐being and happiness but only until depressive symptoms were considered; There was no evidence of an association between PUFAs and life satisfaction. While the literature proposed several mechanisms supporting the possibility that PUFAs may increase well‐being, current results suggest that the relationship was not strong enough to be detected or remained unaffected by covariates. Nevertheless, it is worth taking into consideration the small quantity of PUFAs intake reported in this study, as the absence of statistical significance does not necessarily translate to the relationship being non‐existent, but that the data in this study did not provide sufficient evidence to confidently support a significant association.

Lastly, fish intake was investigated. Interestingly, higher fish intake was associated with higher happiness even when all covariates including depressive symptoms were considered. This supports the literature that proposes a role for omega‐3s (abundant in fish) in modulating healthy dopamine and serotonin levels, also known as the feel‐good or mood‐boosting brain chemicals (Healy‐Stoffel & Levant, 2018; Su et al., 2015; Watson, 2021; Zhou et al., 2022). Fish intake was positively associated with eudemonic well‐being until social factors were considered, as well as with life satisfaction until wealth, education, and ethnicity were considered, suggesting that these covariates contributed to a substantial part of the associations.

As the results are cross‐sectional, the direction of associations cannot be established with confidence. Nonetheless, if it were to be the case that better well‐being leads individuals to eat healthier, we should see consistent results of high F&V, PUFAs, and fish intake in relation to higher positive well‐being. Additionally, if it was simply health behaviour instead of diet or nutrition that influenced well‐being, we should see consistent positive associations across all diets, but this was not the case.

Despite variation in results, novel conclusions can be drawn. First, different types of food are differentially associated with well‐being. For instance, F&V is associated with all domains but strongest with eudemonic well‐being; Fish is associated with happiness independently of depressive symptoms; PUFAs are associated with eudemonic well‐being and happiness to a certain extent, but not life satisfaction. This suggests the possibility that different foods offer different nutritional benefits that may impact psychological health differently. It also suggests the need for further research to investigate what makes specific foods beneficial for psychological well‐being, as the results do not support the likelihood that all healthy diets have the same influence. Furthermore, F&V appears to have the strongest link to positive well‐being compared with other types of dietary intake.

### Strengths

The present study analysed a large, nationally representative sample of ELSA, which increases the generalizability of the study's findings to the broader English population. Furthermore, while past research typically only examined a single diet and well‐being domain, this study examined multiple dietary exposures on three domains of positive well‐being in a large cohort of middle‐aged and older adults in England, adding further knowledge to the existing literature and allowing for a comparison of the relationships between different kinds of diet and well‐being to be observed. The association was explored in a real‐world setting, and as various covariates were added to the models, the relationship between diet and well‐being was investigated more comprehensively. We examined whether the relationship remains despite taking factors known to have a significant impact on well‐being into account and whether the relationship is independent of negative psychological states such as depression. Consequently, this may suggest the strength of the influence certain types of food have on a particular domain of well‐being.

### Limitations

As the study is cross‐sectional and correlational, results should be interpreted with caution as causal effects cannot be assumed. Diet could affect well‐being through processes such as modulation of inflammation and oxidative stress, or alternatively, people with higher well‐being may select healthier and more varied diets. Diet assessment was newly introduced in Wave 9 of ELSA, therefore, the direction of association cannot be examined. Longitudinal or experimental research would help clarify the direction and underlying mechanisms of the relationship.

As most ELSA data were self‐reported, this may introduce recall and reporting biases, along with subjectivity, potentially undermining true results. However, this was addressed by using measurements with strong psychometric properties or previously validated in similar populations to minimize error. While future studies could measure intake objectively (e.g., assigned diet, weighing/controlled amount), well‐being is inherently subjective in nature. Moreover, while objective measures provide precision, they may not represent usual consumption.

### Implications

The current study contributes to this field of research by advancing the knowledge of whether diet influences positive well‐being, which could inform lifestyle adjustments or interventions. The novelty is a currently limited focus on positive psychology and an investigation that includes various types of dietary intake and well‐being domains where comparisons can be observed. As ageing is often accompanied by a health decline, an effort in prevention in addition to treatment regarding the health and well‐being of middle‐aged and older adults could help reduce costs to the individuals, families, and healthcare systems, as well as increase the opportunity for a long, healthy life.

With diet being a modifiable risk factor, improvements may be beneficial for psychological well‐being and consequently better health and ageing, consistent with past evidence that suggested a protective role of psychological well‐being on health, ageing, and survival. Identifying predictors of well‐being helps inform beneficial lifestyle adjustments, behaviour changes, or dietary interventions. Practical implications include increasing consumption of F&V, fish, and foods high in PUFAs (e.g., nuts and seeds, fatty fish). Government or private sectors can provide support by making healthy diets more accessible and affordable, possibly through funding or building programmes to provide those foods for the target population, and lower pricing for relevant products. This aligns with previous research conclusion, which suggest increasing F&V consumption as a simple, cost‐effective way to improve the health and quality of life of older adults (Gehlich et al., 2019). It is important to reiterate that correlational relationships do not establish causation, and while these are plausible implications, further research is needed to test these assertions.

## CONCLUSIONS

The investigation of positive psychological benefits from diet is at an early stage. In this study, positive relationships between diet and positive well‐being were found, although results were attenuated when adjusted for covariates and some were no longer robust when certain factors such as depressive symptoms were considered. Results varied between different diets and domains of positive well‐being, suggesting the influence different diets have on well‐being and encouraging behaviour change towards consuming more F&V, PUFAs, and fish. Further longitudinal and experimental research is needed for a stronger conclusion to be reached.

## AUTHOR CONTRIBUTIONS

**Pepper Thanaporn Theeraoat:** Conceptualization; investigation; writing – original draft; methodology; validation; visualization; writing – review and editing; software; formal analysis; project administration; data curation; resources. **Ruth A. Hackett:** Writing – review and editing; supervision; visualization. **Joseph Chilcot:** Writing – review and editing; supervision. **Andrew Steptoe:** Writing – review and editing; supervision; conceptualization; methodology; resources; data curation.

## CONFLICT OF INTEREST STATEMENT

The authors declared no conflicts of interest.

## Data Availability

The data that support the findings of this study are primarily available through the UK Data Service (UKDS) at https://doi.org/10.5255/UKDA‐SN‐5050‐32.
